# The roles of fungus in CNS autoimmune and neurodegeneration disorders

**DOI:** 10.3389/fimmu.2022.1077335

**Published:** 2023-01-26

**Authors:** Chuyu Wu, Mei-Ling Jiang, Runqui Jiang, Tao Pang, Cun-Jin Zhang

**Affiliations:** ^1^ Nanjing Drum Tower Hospital Clinical College of Traditional Chinese and Western Medicine, Nanjing University of Chinese Medicine, Nanjing, Jiangsu, China; ^2^ State Key Laboratory of Natural Medicines, Jiangsu Key Laboratory of Drug Screening, Jiangsu Key Laboratory of Drug Discovery for Metabolic Diseases, China Pharmaceutical University, Nanjing, China; ^3^ Department of Neurology, Nanjing Drum Tower Hospital, Medical School and the State Key Laboratory of Pharmaceutical Biotechnology, Nanjing University of Chinese Medicine, Nanjing University, Nanjing, Jiangsu, China; ^4^ Institute of Brain Sciences, Institute of Brain Disorder Translational Medicine, Nanjing University, Nanjing, Jiangsu, China; ^5^ Jiangsu Key Laboratory for Molecular Medicine, Medical School of Nanjing University, Nanjing, Jiangsu, China; ^6^ Jiangsu Province Stroke Center for Diagnosis and Therapy, Nanjing, Jiangsu, China

**Keywords:** fungus, brain disorders, multiple sclerosis, signaling, disease

## Abstract

Fungal infection or proliferation in our body is capable of initiation of strong inflammation and immune responses that result in different consequences, including infection-trigged organ injury and inflammation-related remote organ dysfunction. Fungi associated infectious diseases have been well recognized in the clinic. However, whether fungi play an important role in non-infectious central nervous system disease is still to be elucidated. Recently, a growing amount of evidence point to a non-negligible role of peripheral fungus in triggering unique inflammation, immune response, and exacerbation of a range of non-infectious CNS disorders, including Multiple sclerosis, Neuromyelitis optica, Parkinson’s disease, Alzheimer’s disease, and Amyotrophic lateral sclerosis et al. In this review, we summarized the recent advances in recognizing patterns and inflammatory signaling of fungi in different subsets of immune cells, with a specific focus on its function in CNS autoimmune and neurodegeneration diseases. In conclusion, the fungus is capable of triggering unique inflammation by multiple mechanisms in the progression of a body of CNS non-infectious diseases, suggesting it serves as a key factor and critical novel target for the development of potential therapeutic strategies.

## Introduction

Fungal infection or proliferation in our body is capable of initiation of strong inflammation and immune responses that result in different consequences, including infection trigged organ injury and inflammation related remote organ dysfunction. Normally, only a very small percentage of fungi can cause human infections. It has been known that the incidence of fungal infections of the central nervous system (CNS) increased significantly in the past few years (1, 2). This is possibly due to the widely use of immunosuppressive therapies (e.g., chemotherapy or corticosteroid treatment), the stem cells and organ transplants, and the spread of HIV/AIDS (3). There are various clinical manifestations, mainly meningitis, encephalitis, hydrocephalus, brain abscess, and stroke syndrome. The pathogens of CNS fungal infections include yeasts, dimorphic fungi, and filamentous fungi (1). *Cryptococcus neoformans*, *Aspergillus*, and *Candida albicans* are the most common pathogens. Fungi usually enter the human body through the respiratory tract and spread to the CNS *via* the bloodstream. Immunocompetent individuals can also become infected as a result of surgery, trauma, intravenous drug use, or contaminated medical supplies. Fungi are usually disseminated to the CNS from adjacent tissues (sinusoid structures, mastoid processes, or orbital orbits) (3).

The blood-brain barrier (BBB) prevents chemicals and germs from passing through, thereby safeguarding the CNS. *C. albicans* crosses the BBB through both the in-paracellular pathway and the transcellular pathway (4). More evidence implicates that *C. albicans* enter the brain through the transcellular pathway. *C. albicans* can cross the BBB by binding to the heat shock protein gp96 on microvascular endothelial cells (5). and this process requires the interaction of the invasin Als3 with gp96 to induce fungal endocytosis.

Cryptococci also cross the BBB in multiple ways. After initially adhering to the BBB, Cryptococcus neoformans forms biofilm-like cell clusters on the brain unit. Such aggregated cells are able to penetrate the BBB without severely affecting endothelial cells tight junctions or forming pores in the layer of endothelial cells (6). The “Trojan horse” approach by Cryptococcus to infiltrate the brain has been demonstrated by *in vitro* experiments that phagocytes holding engulfed viable *C. neoformans* to pass the endothelial cell layer of the brain (7, 8). Additionally, *Aspergillus fumigatus* mycotoxin gliotoxin penetrates and damages the human blood-brain barrier *in vitro (* 9).

In addition to fungal-associated infectious diseases, recent advances have also suggested a remarkable role of fungi in several non-infectious autoimmune diseases and neurodegeneration diseases, such as Multiple sclerosis (MS), Neuromyelitis optica spectrum disorder (NMOSD), Alzheimer’s disease (AD), Parkinson’s disease (PD), Amyotrophic lateral sclerosis (ALS). While the classic pathogenic feature of MS is the presenting of the death of oligodendrocyte and CNS demyelination, an outcome of neuron death has believed to be the cause of AD, PD, and ASL. Of note, the common characteristics of these non-infectious diseases are sterile inflammation that takes place in CNS. However, whether fungi play a critical role in these disorders has been unclear. Here, we outline recent studies on the role and mechanisms of fungi in these non-infectious CNS autoimmune and neurodegeneration disorders.

## PRRs and CARD9 signaling in fungi recognition

Although Fungi infection or proliferation is a common event, the population of fungi is usually efficiently cleared or suppressed under an intact immune system and homeostasis condition. The fungi-triggered immunity includes both the innate and adaptive immune systems. In fungal infections, the innate immune response plays an important role in preventing the onset of fungal infection, while the adaptive immune response is closely associated with recovery from fungal disease. Innate immunity constitutes the first line of host defense, which includes physical barriers such as skin and mucosa, the complement system, antimicrobial peptides (AMPs), and cell-mediated protection, recognizing and initiating an inflammatory response to invade or inhibit the fungi populations.

**Figure 1:**
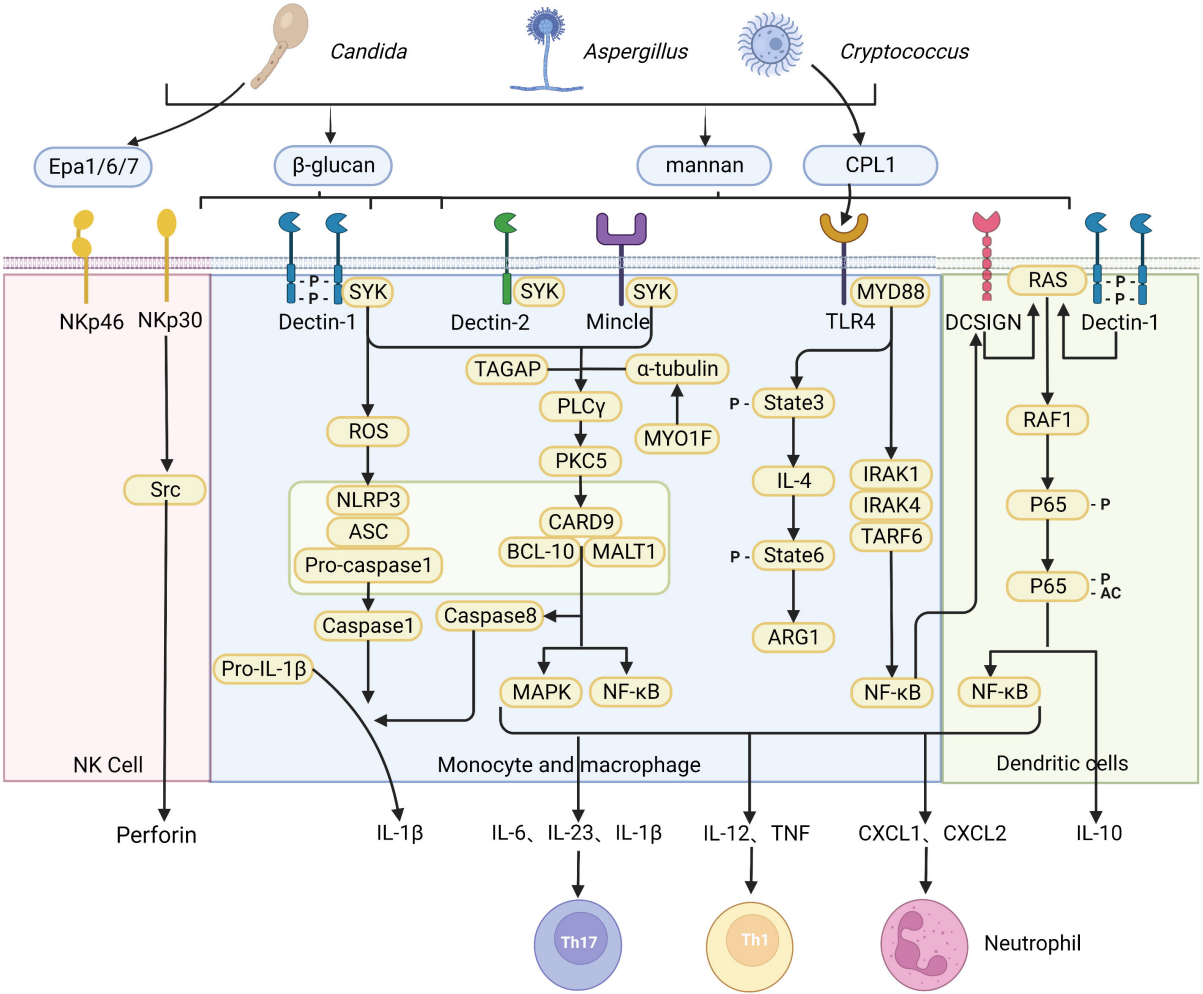
Recognition of fungi by pattern recognition receptors. A diagrammatic representation of pattern recognition receptors involved in the recognition of various fungal species, and the cellular responses triggered by ligand-receptor binding. The CLR family of receptors recognize fungal carbohydrates and result in activation of the SYK–CARD9 axis, formation of the CARD9–BCL10–MALT1 complex and activating nuclear NF-κB and MAPK signaling pathways, which in turn activate synthesis and release of pro-inflammatory cytokines and chemokines including IL-6, IL-1β, IL-23, IL-12, CXCL1 and CXCL2. The release of chemokines and cytokines that promote CD4^+^ T cell differentiation into specific T helper cell. The NOD-like receptor NLRP3 forms inflammasome complex with ASC and caspase-1 that results in production of IL-1β. In addition, dectin-1 signaling engages the CARD9–BCL10–MALT1 complex to promote non-canonical inflammasome activation that results in IL-1β production *via* caspase-8. In contrast, NK cells equipment with receptor NKp30 and NKp46 as a microbial pattern-recognition receptor to recognize fungi. C-type lectin DC-SIGN modulates TLR signaling *via* Raf-1 kinase-dependent acetylation of NF-κB in DCs. (Created with Biorender.com).

Activation of innate immune response relies on a lineage-encoded receptor, also known as a pattern recognition receptor (PRR), which recognizes a variety of pathogen-associated molecules (pathogen-associated molecular patterns, PAMPs) expressed by invading microorganisms. PRRs are primarily expressed by antigen-presenting cells (APCs), including dendritic cells (DC) and macrophages. PAMPs are known to play a crucial function in pathogens-cell interaction and are often shared by whole classes of microorganisms. In addition, PAMPs are either structural determinants or necessary for pathogenicity. Both Candida and Aspergillus undergo a constant morphological switch between yeasts and hyphae, resulting in variable fungal PAMPs exposure. The cell wall of *C. albicans* contains carbohydrate polymers and glycoproteins, including chitin, β-1,3- and β-1,6-glucans, and N- and O-linked mannan (10). In contrast, the Aspergillus cell wall is mainly composed of chitin, α-glucans, β-1,3-1,4-glucans, and galactomannans. An important feature of the Aspergillus conidial cell wall is the hydrophobic rodlets in outer layer. During swelling of the conidial cell wall and subsequent germination, the hydrophobic layer changes into a hydrophilic structure exposing polysaccharides. Different from *C. albicans* and Aspergillus, the cell wall of Cryptococcus is composed of glucan, chitin, chitosan, mannoprotein and pigment melanin. Of note, its cell wall is wrapped with an exopolysaccharide capsule, which masks the available PAMPs in the cell wall, resulting in limited recognition of Cryptococcus by many PRRs (11). During fungal infection and invasion, the first critical step for an effective immune response is the recognition of invaders. Various PRRs expressed on different cells are competent in recognizing fungal invaders and trigger effective signaling pathways and cellular responses (**Figure 1**).

Among different pattern recognition receptors (PRRs), C-Type lectin receptors (CLRs) are the most common receptors that recognize fungi, while Toll-like receptor (TLR) and Nod-like receptor (NLR) play a secondary role. The Members of the CLR superfamily include Dectin-1, Dectin-2, macrophage C-type lectin, macrophage-induced C-type lectin, mannose receptor, melanin-sensing C-type lectin, and dendritic cell-specific intercellular adhesion molecule-3-grabbing nonintegrin (12). CLRs recognize fungal carbohydrates such as β-glucans, α-mannan, hyphal mannose, glycolipids, and chitin that are present within the cell wall (13), but can also recognize other components such as melanin (14). Recognition of fungal cell wall components by various CLRs leads to activation of splenic tyrosine kinase (Syk) in DC and macrophages (15), triggering assembly of the CARD9-BCL10-MALT1 (CBM) complex, which further leads to activation of nuclear factor-κB (NF-κB) and mitogen-activated protein kinase (MAPK) signaling pathways, which in turn activates synthesis and release of pro-inflammatory cytokines and chemokines including IL-6, IL-1β, IL-23, IL-12, CXCL1, CXCL2 and GM-CSF, among others. CLR intracellular signaling pathways promote maturation and migration of DC to the draining lymph nodes to activate naïve T cells. This process is dependent on the release of DC-derived chemokines and cytokines that promote CD4^+^ T cell differentiation into various T helper cell subsets, including Th1, Th2, Th9, Th17, and regulatory T cells (Tregs). Besides, the antifungal adaptive T cell immunity is also activated at the same time.

**Figure 2:**
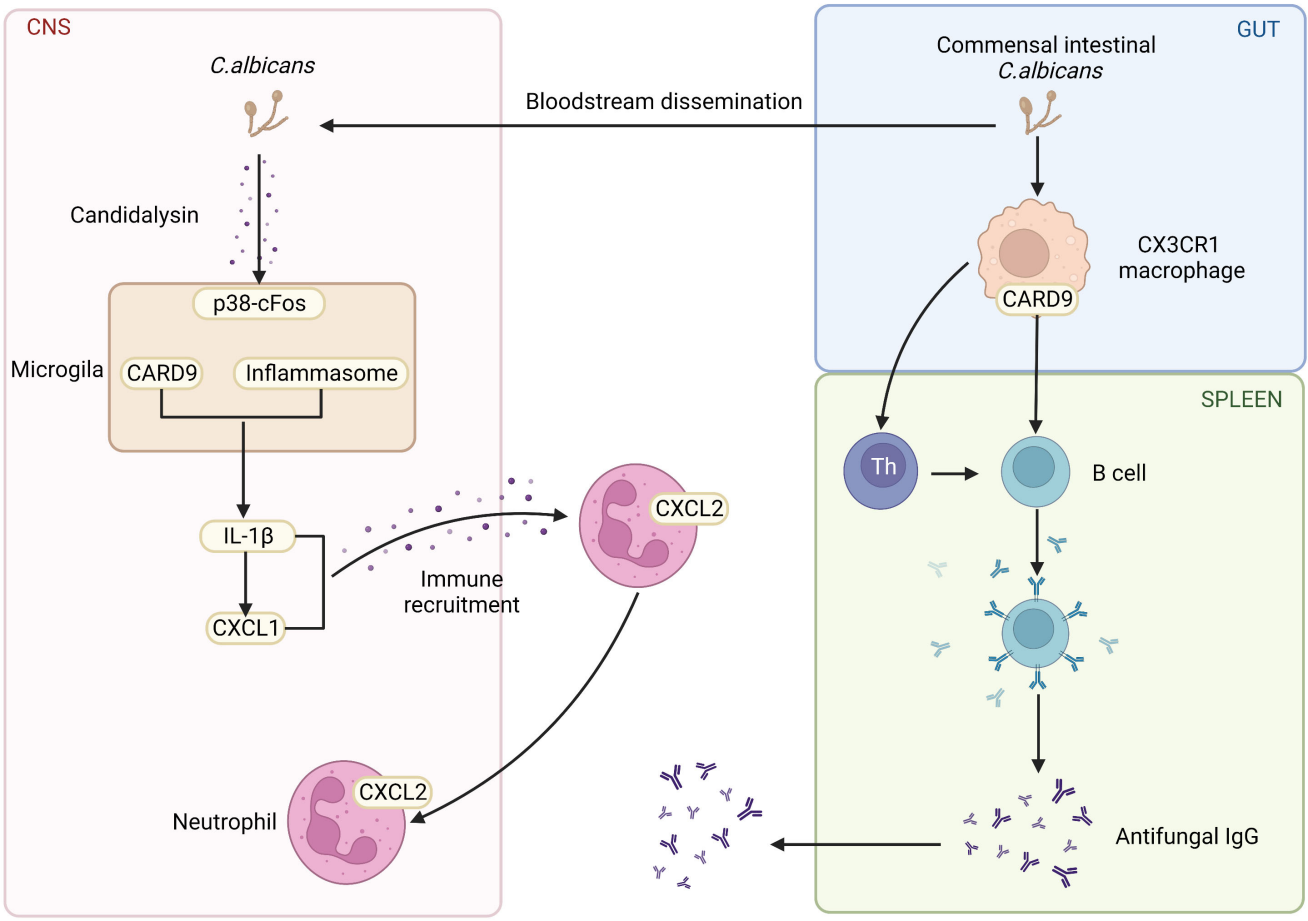
The role of CARD9 pathway in the brain and gut. Commensal Intestinal *C. albicans* induce the production of antifungal IgG. CARD9 and CX3CR1 double-positive macrophages promote the generation of antifungal IgG. CARD9-deficiency increases the susceptibility of fungal-specific CNS-targeted infection. In the fungal-infected CNS, microglia rely on CARD9 for producing IL-1β and CXCL1, which recruits neutrophils to clear the fungi. (Created with Biorender.com).

Others and our groups have shown that CARD9 is crucial for maintaining gut homeostasis with commensal organisms and preventing opportunistic fungi from invading tissue. One of the crucial pieces of evidence is that the abnormalities of the CARD9 pathway often result in the increase of the fungi population, even severe fungi infections. Moreover, autosomal recessive CARD9 deficiency in human leads to profound defects in the production of pro-inflammatory cytokines in response to stimuli specific to fungi, including IL-6, IL-1β, GM-CSF, and TNFα (16). Similar results are also confirmed in animal model. It has been shown that mice with CARD9 genetic deletion produce less pro-inflammatory cytokines, which impairs myeloid cell survival after exposure to *C. albicans*, a significant inducer of antifungal immunoglobulin G(IgG) (17). Furthermore, CARD9 and CX3CR1 double-positive macrophages regulate innate immunity and are necessary for the generation of antifungal IgG (**Figure 2**) **(** 18). In contrast to the critical role of CARD9 in peripheral, CARD9 also engages the protective antifungal immunity within the CNS. It was reported that human CARD9 deficiency causes fungal-specific CNS-targeted infection susceptibility (**Figure 2**). In all, PRRs and CARD9 signaling play indispensable roles in fungi-mediated immunity.

## The T cells associated immune responses after fungi recognition

CD4^+^ T cells are thought to play an indispensable role in fungi-triggered immune responses. While this response contributes to the recovery of both superficial and invasive fungal infections, an overactive immune response could also result in or aggregate the development of several diseases. Unique T cell responses are aggressively induced in response to lysates and peptides of *C. albicans* or *A. fumigatus*, delivered by autologous monocytes. The differentiation and activation of various T cell subsets depend on the activities of APCs which recognize fungi through CLRs. Among the T cell subsets, Th1 cells secrete IFN-γ, GM-CSF, and TNF, which affect the maturation and killing capacity of phagocytes and the function of APC. TNF synergizes with IFN- to cause the generation of ROS of macrophage *in vitro*, which is hypothesized to be a factor in the growth arrest of intracellular fungal infections such as *Coccidioides immitis* and *Histoplasma capsulatum in vivo* (19, 20). As another T cell subset in addition to Th1, Th17 cells release specific signature cytokines including IL-17A, IL-17F, and IL-22 that aid in fungicidal activity, neutrophil trafficking, and the activation of AMPs from epithelial and keratinocyte cells protect against fungal overgrowth (21). In mice, Th17 cells have also been demonstrated to promote the production of Immunoglobulin A (IgA), an immunoglobulin that is crucial for protection and homeostasis at mucosal surfaces (22, 23). Moreover, Th17 cells support host immunological homeostasis against microbiota, at least in part, by inducing epithelial pIgR expression through IL-17, which boosts IgA secretion into the lumen (23). Of note, Th17 cells are capable of converting into T follicular helper cells (TFH), which may be essential for inducing the development of IgA-producing B cells (22). Thus, Candida-specific Th17 cells may play an essential role in fungal intestinal homeostasis through their ability to convert into TFH cells. In addition, studies have also shown that the gut mycobiome may be involved in providing protection against other pathogens at distal sites. For instance, Th17 cell responses in humans are specifically induced by *C. albicans*. Cross-reactivity to *C. albicans* causes the production of Th17 cells that attack other fungi. Both *C. albicans* and cross-reactive Th17 cells are increased by intestinal inflammation (24). Numerous novel molecules involved in antifungal signaling pathways have been recently identified by others and our group, such as MYO1F (25), STIM1 and TRIM31 (**Table 1**). Several antifungal drugs have also been developed based on newly discovered antifungal molecules (**Table 2**).

**Table 1:** Antifungal signaling pathways discovered in the last 5 years.

Target	Mode of action	Spectrum of activity
TRIM31 (26)	TRIM31 interacts with SYK and catalyzes polyubiquitination of SYK. Lack of TRIM31 in bone marrow-derived dendritic cells and macrophages inhibits SYK-mediated signaling pathways.	*C. albicans*
MYO1F (25)	MYO1F recruit adaptor protein AP2A1 to promote fungus-induced acetylation of α-microtubulin, and acetylated α-microtubulin promotes Syk and card 9 translocation from the plasma membrane to the cytoplasm to activating antifungal signaling.	*C. albicans*
Dok3 (27)	downstream of kinase 3 (Dok3) recruits protein phosphatase 1 (PP1) to dephosphorylate Card9, suppressing Card9 signaling.	*C. albicans*,
Commensal *C. albicans (* 28)	intestinal colonization with *C. albicans* drives systemic expansion of fungal-specific Th17 CD4 T cells and IL-17 responsiveness by circulating neutrophils, which synergistically protect against *C. albicans* invasive infection.	*C. albicans*,
STIM1 (29)	STIM1 deletion in all immune cells increased susceptibility to *C. albicans* infection in the mucosa. STIM1 deletion reduced the production of Th17 cytokines, which are important for antifungal immunity, as well as the expression of genes involved in various metabolic pathways, including Foxo and HIF1 signaling, which regulate glycolysis and oxidative phosphorylation.	*C. albicans*
Kupffer cells (30)	Kupffer cells in the liver have a prominent function in the capture of circulating Cryptococcus neoformans and *C. albicans*, thereby reducing fungal dissemination to target organs.	*C. neoformans* and *C. albicans*
Xp38γ and p38δ (31)	p38γ and p38δ regulate the AK1-TPL2-MKK1-ERK1/2 pathway in macrophages, which is activated by Dectin-1 splicing. In mice, p38γ/p38δ deficiency increases the antifungal capacity of neutrophils and macrophages by increasing the production of ROS and iNOS.	*C. albicans*
fungal melanin (14)	fungal melanin is an essential PAMP required for the Warburg shift and the ensuing immunometabolic responses in macrophages.	*C. albicans* and *A. fumigatus*
antifungal IgG (18)	The intestinal commensal fungus *C. albicans* is a major inducer of antifungal IgG. B cells expand in extraintestinal lymphoid tissue and produce systemic antibodies to prevent disseminated fungal infections. Production of antifungal IgG is dependent on CARD9 and CARD9CX3CR1 macrophages.	*C. albicans* and *C. auris*
Dock2 (32)	After fungal stimulation, activated SYK phosphorylates DOCK2 to promote the recruitment and activation of RAC GTPase, which then increases ROS production and downstream signaling activation to clear the fungus intracellularly.	*C. albicans*
JNK1 (33)	JNK1 activation suppresses anti-fungal immunity in mice. NK1 deficiency leads to significantly higher induction of CD23, a novel C-type lectin receptor, through NFATc1-mediated regulation of the CD23 promoter.	*C. albicans*

**Table 2:** Antifungal drugs discovered in the last 5 years.

drug	therapeutic target	Effect of administration	Spectrum of activity
Sirt2 deacetylase inhibitors (AGK2, AK-1, or AK-7) (25)	Inhibition of the deacetylase SIRT2 enhances α-tubulin acetylation and thus enhances antifungal immune responses at the cellular level.	administration of the Sirt2 deacetylase inhibitors AGK2, AK-1 and AK-7 protected mice against both systemic and central nervous system *C. albicans* infections.	*C. albicans*
Nanocarrier-delivered Rac1 mRNA(mRac1@LNP) (32)	enhancing antifungal immunity by boosting Rac1 activity through the introduction of nanocarrier-delivered Rac1 single-stranded mRNA. mRac1@LNP transfection significantly increased ROS production after curdlan, mannan or HKCA stimulation.	injection of mRac1@LNPs significantly attenuated the severity of candidemia.	*C. albicans*
JNK1 inhibitor(SP600125, JNK-IN-8) (33)	Increased CD23 expression is induced when jnk1 is inhibited, which promotes antifungal immunity.	JNK inhibitors exerted potent anti-fungal therapeutic effects in *C. albicans*-infected mouse and human cells.	*C. albicans*

In addition to T cell subpopulations, microglia and natural killer (NK) cells also play important roles in fungi immunity partially by collaboration with CD4^+^ T cell. Microglia exhibit strong responses to various species of fungi, including C. neoformans and *C. albicans* (34, 35), utilizing the receptor GPR43 (36), PI3 kinase signaling (37), and inducible nitric oxide synthase (iNOS) (38) for C. neoformans phagocytosis and killing. Moreover, there are other APCs in the brain, such as astrocytes and recruited DCs (38), that may also be involved in the induction of specific T-cell immunity and CD4^+^ T migration to the brain through an increase in TNF, CCL2, and CCL5 in experimental cryptococcosis (39). In the fungal-infected CNS, microglia is capable of producing IL-1β and CXCL1 by the fungal-secreted toxin Candidalysin, which recruits neutrophils to clear the fungi (40, 41). Besides, NK cells are capable of using the activating receptor NKp30 as a microbial PRR to recognize fungi and activate cytolytic pathways. NKp30 recognizes β-1,3-glucan, which induces Src family kinase signal transduction, cytotoxic granule trafficking, and synapse formation (42). NKp46 is another kind of NK activating receptor, which recognizes the Epa adhesins (43). Although fungi-associated immunity is complex, fine-turning of the overactive immune response is important to control the infection and reduce inflammatory injury.

## Fungi in CNS autoimmune and neurodegeneration disorders

### Multiple sclerosis

Multiple sclerosis (MS) is a prototypical autoimmune neuroinflammatory disease of the CNS characterized by multifocal demyelination and neurodegeneration. The autoimmune response of MS is pathologically characterized by pro-inflammatory cell infiltration in the CNS, initiated by Th1/Th17 cells and promoting the proliferation of innate immune cells and B cells. The onset of MS is thought to be caused by the activation of peripheral CNS reactive T cells (44). The two main hypotheses are the identification of immune cross-reactivity of T cells with foreign antigens and the leakage of CNS-derived antigens into deep cervical lymph nodes. The etiology of this incurable disease is unknown. Many microbes have been investigated as potential causes of MS, such as the Epstein Barr virus and some bacteria (45). In recent years, Genome-wide association studies have identified several antifungal immune genes associated with autoimmune disease (46, 47). Significant enrichment of MS susceptibility loci is evident in many different immune cell types and tissues, but lack of enrichment in the CNS profiles at the tissue level. Although the adaptive immune system has been proposed to play a significant role in MS pathogenesis (48), analysis of the genetic map of MS patients demonstrates that many elements of innate immunity, such as NK cells and DCs, also show strong enrichment of MS susceptibility genes (48). These findings provide a genetic link between innate immune dysregulation and susceptibility to autoimmune disease. However, the mechanisms by which dysregulation of innate immune signaling pathways leads to autoimmune disease are unclear.

Recent studies and our group have also suggested that fungi may play an important role in the pathogenesis of MS (25, 49, 50). It has been reported that fungi can be detected in the brain specimens of MS patients by using nested PCR assays and next-generation sequencing. Among fungal DNA of different species, *Trichosporon mucoid* was found in the majority of MS patients (51), and Candida is the most closely related to multiple sclerosis as reported (52–54). *C. albicans* isolated from MS patients had higher specific enzymatic activity and were positively correlated with the severity of MS (52). In addition, MS patients had a higher rate of candida infection than the healthy controls (51). For example, the HLA-DRB1*15 allele group is the most important genetic risk factor for multiple sclerosis (55) and is also a risk factor linked to fungal infections (56). The evidence supported that Candida species infection may be associated with increased odds of MS. It reported that *C. albicans* infection before the induction of experimental autoimmune encephalomyelitis (EAE), the classic animal model of MS, promotes the EAE onset and aggravates the disease severity (57). In addition to *C. albicans*, EAE was also aggravated in mice infected with non-*C. albicans* strains *C. glabrata* and *C. krusei (* 58), suggesting a critical role of fungi in promoting EAE disease progression.

Although the mechanism by which immune cells are directed into the CNS is unknown, it is believed that activation of peripheral T cells triggers the expression of adhesion molecules and chemokines, thereby facilitating the migration of immune cells. MS lesions show increased expression of endothelial adhesion molecules such as intercellular adhesion molecule 1 (ICAM-1), vascular cell adhesion molecule 1 (VCAM-1), and dual immunoglobulin domain-containing cell adhesion molecule (DICAM) (59, 60). These adhesion molecules and their ligands promote T cell migration in the BBB. In VLA-4-deficient mice, auto-reactive T cells fail to migrate to the CNS (61). adhesion molecule DICAM expression is increased on circulating CD4 T cells in patients with active RRMS and PMS disease course, while expression of ligands of DICAM is increased in BBB endothelium and MS lesions. DICAM promotes Th17 crossing during autoimmune neuroinflammation in the BBB (60).

**Figure 3:**
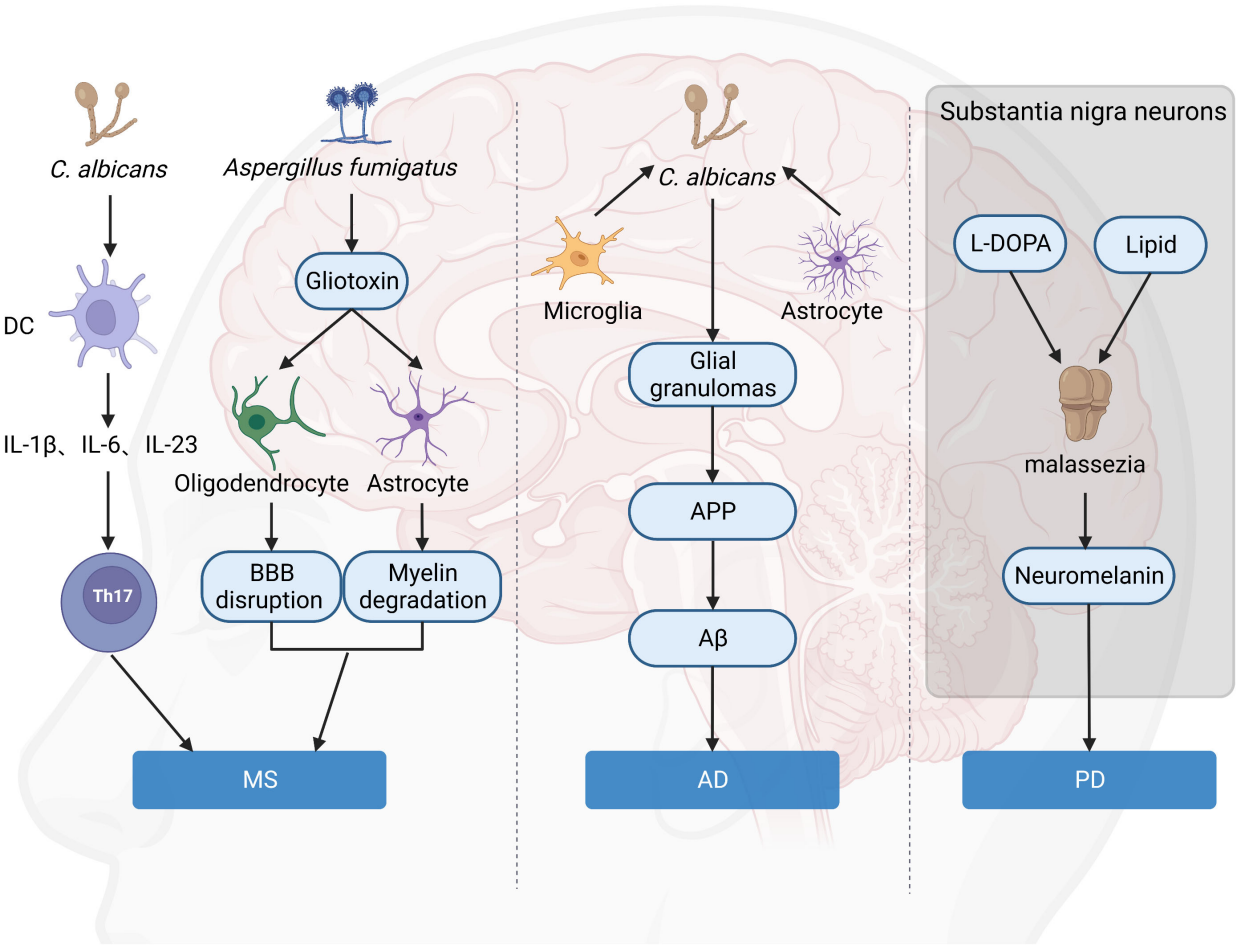
Hypothesis of the role of fungi in brain disorders. Gliotoxin attacks on astrocytes and oligodendrocytes around the BBB lead to BBB destruction and myelin degradation, which aggravates MS. C. albicans is a significant inducer of human Th17 cells, and TH17 exacerbates MS. C. albicans infection of the brain causes activated microglia and astrocytes to accumulate around yeast aggregates, forming fungal-induced glial granulomas. APP aggregates at the periphery of these granulomas, and APP cleavage leads to the production of Aβ. Malassezia consumes intracellular lipid droplets of neurons in L-DOPA-rich substantia nigra and produces neuromelanin, leading to exacerbation of PD. (Created with Biorender.com).

It has been proposed that fungal toxins may play a role in the destruction of astrocytes and oligodendrocytes, resulting in characteristic myelin degradation (62). Gliotoxin is the main and the most potent mycotoxin that is secreted by *A. fumigatus*. Gliotoxin may contribute to the cerebral invasion processes of Aspergillus fumigatus by altering the BBB integrity (9). Once the toxin reaches the BBB, astrocytes, and oligodendrocytes surrounding the BBB will become targets for attack. Astrocytes play an integral role in maintaining barrier integrity, while oligodendrocytes provide nutritional maintenance for myelin. The result includes disruption of the BBB, enhanced CNS inflammation, degradation of myelin, and exacerbation of MS (**Figure 3**). This hypothesis is also supported by the fact that Gliotoxin can increase neuroinflammation of EAE in non-neuronal sites (63). In addition, chitinase can hydrolyze chitin, a component of fungal cell wall but not present in bacterial and mammalian cells, while elevated chitinase in cerebrospinal fluid is an important biomarker of MS (64, 65). Besides, calprotectin is elevated in the cerebrospinal fluid of MS patients, and amyloid deposits are found in the brains of MS patients (66) (67), both of which are associated with antifungal immunity, suggesting fungal infection in the CNS of MS patients is a critical event for disease.

Although fungi have been detected in the CNS of MS patients, attacks on the immune system by fungi in the periphery may also exacerbate MS. ITS sequencing of MS patients and healthy controls showed that Aspergillus and Saccharomyces were significantly increased in the gut microbiome of MS patients (68). Alterations in the gut mycobiome in MS patients lead to changes in various types of immune cells. Aspergillus are positively correlated with B cells and activated CD16 DCs in MS patients, while Saccharomyces was positively correlated with circulating basophils and negatively correlated with regulatory B cells (68). Immune cells infiltrating in the CNS of MS are thought to be initiated in the periphery and reactivated in the CNS by local APCs (69). Many drugs for MS work through this. For example, alemtuzumab and ocrelizumab work by depleting peripheral immune cells, and natalizumab works thought blocking cell migration (44). Foreign antigens in tissues such as intestine, lung, and skin are captured by APCs, which migrate to draining lymph nodes and may activate pathogen-reactive T cells that cross-react with CNS self-antigens (70). After they are activated and polarized into pro-inflammatory cells, these self-reactive T cells migrate to the CNS, causing tissue damage.

The antifungal immunity and the immune response to exacerbate MS largely overlap, leading to speculation that fungi be responsible for the onset or exacerbation of MS (71). While Th17 cells are involved in the pathogenesis of several autoimmune diseases, including MS (72), TH17 cells also play an important role in antifungal immunity (73). Our recent studies suggested that fungi activate the peripheral macrophage in an EPHB2 pathway and lead to the secretion of robust inflammatory cytokines, such as IL-23, IL-12, and IL-1β, which directly promote the activation of peripheral Th1 and Th17 (50). As a critical receptor, Dectin-1 plays an important role during the above process. Dectin-1 is capable of inducing the production of IL-1β, which directly activates Th17 cellular response during fungal infections *via* CARD9- and NF-κB signaling. According to recent research by M. Elizabeth Deerhake, Dectin-1 and its downstream signaling molecule CARD9 not only played a role in fungal infection but were also involved in EAE pathogenesis. Of note, Dectin-1 inhibits EAE progression, whereas CARD9 play the opposite role. Dectin-1 promotes myeloid cell-astrocyte crosstalk in EAE by upregulating Oncostatin M through a CARD9-independent mechanism, suggesting an important function of Dectin-1 in EAE disease (74). Human carrying TAGAP mutation has increased susceptibility to fungi infection. Similarly, mice lacking TAGAP mount inefficient innate and adaptive immune responses featured with impaired cytokine release by macrophage and Th17 cell differentiation during *C. albicans* infection. TAGAP-defective mice exhibit significantly attenuated onset and severity in the EAE model. TAGAP is phosphorylated by EPHB2 at tyrosine 310 in macrophage after stimulation with Dectin ligands, which bridges proximal Dectin-induced EPHB2 activity to downstream CARD9-mediated signaling pathways (50). The current pieces of evidences suggest that fungi may promote MS *via* direct invasion to CNS or regulation of the peripheral T cells, which could migrate to the brain and promote the disease progression, suggesting fungi signaling may be the possible target for MS treatment.

### Neuromyelitis optica

Neuromyelitis optica spectrum disorders (NMOSD) are a group of CNS autoimmune diseases characterized by recurrent episodes of optic neuritis and long-segment transverse myelitis (75). In 2004, the discovery of IgG1 against aquaporin 4 (AQP4) made NMOSD a separate disease entity from MS (76). AQP4 antibodies were clinically detectable in the serum and cerebrospinal fluid of approximately 70%-80% of NMOSD patients. The exact cause of NMO is not yet known. However, most experts believe that NMO is caused by dysfunction of immune tolerance, which refers to the ability of the immune system to distinguish its own cells or proteins from potentially foreign substances. It is believed that self-reactive T and B cells, autoantibodies, complement and leukocytes play a role in NMO (77).

The pathogenesis of NMO is influenced by a combination of genetic and environmental factors, including infectious agents. Several infectious diseases can trigger or exacerbate autoimmunity in NMO. Although there is no direct evidence linking NMO to fungal infections, some studies have shown that *C. albicans* can slightly promote the degree of lymphocyte proliferation in PBMC of NMO patients, suggesting that fungal infections may also exacerbate NMO (78).

### Alzheimer’s disease

Alzheimer’s disease (AD) is a neurodegenerative disorder characterized by the presence of amyloid beta (Aβ), accumulating extracellularly as amyloid plaques and phosphorylation of tau proteins, and neurofibrillary tangles (NFTs) in the brain, affecting neuronal connectivity and functioning, which results in a progressive loss of brain function (79). Studies have shown that intestinal colonization of mucosal fungi induces the systemic release of IL-17A, which increases mouse responses to social stimuli by mediating the IL-17R pathway in neurons (80). AD-related chronic neuroinflammation is manifested by the activation of microglia to produce pro-inflammatory cytokines and chemokines, including IL-1β, IL-6, TNF-α, CC motif chemokine ligand-5 (CCL5), and macrophage inflammatory protein-1α (MIP-1α) by TLR2, TLR4 and inflammasome NALP3 signals (81, 82).

Although the cause of Alzheimer’s disease is still uncertain, multiple studies have shown a relationship between microbial infections and AD. Pathogens such as HSV-1, EBV, *Chlamydia pneumoniae*, and *Helicobacter pylori* have been detected in serum, cerebrospinal fluid (CSF) and brain tissues of AD patients (83). Extensive studies have detected the presence of different fungal species or fungal proteins in the brains of AD patients (84–86). Chitin is a N-acetylglucosamine polymer that is found in the fungal cell wall. Chitin-like structures have been identified in Alzheimer’s disease brains by fluorochrome dye calcofluor (87). Others created and tested specific antibodies that detect the fungal proteins enolase and -tubulin and show the presence of two fungal proteins, enolase and α-tubulin, as well as the polysaccharide chitin, in brain tissue of AD patients (86). Control subject brain slices were typically negative for staining with the three antibodies. Furthermore, levels of chitinase, a human enzyme generated by macrophages, are pretty high in the serum and CSF of AD patients. These data support the concept that AD may be caused by a disseminated fungal infection.

With the discovery of the antimicrobial properties of Aβ against fungal and bacterial infections (88), a new AD hypothesis has emerged, called the “antimicrobial protection hypothesis” (89) (**Figure 3**). This hypothesis suggests that the deposition of Aβ plaques in the brain can initiate an early innate immune response. Under normal conditions, Aβ can entrap and neutralize invading pathogens. However, this pathway is overactivated in AD, leading to persistent inflammation, neurodegeneration, and Aβ plaque deposition. Intravenous injection of small numbers of *C. albicans* cells results in highly localized encephalitis characterized by the accumulation of activated microglia and astrocytes around the aggregated fungus, forming fungal-induced glial granulomas. Amyloid precursor protein (APP) accumulates at the periphery of these granulomas, and the cleavage of APP leads to the production of Aβ. *C. albicans* in the CNS activates the transcription factor NF-κB and induces the production of IL-1β, IL-6, and tumor necrosis factor, further upregulating the excessive synthesis of Aβ. Mice infected with *C. albicans* exhibit mild memory impairment, which resolves after fungal clearance (90). All of this evidence suggested a direct connection between fungi and AD onset and progression.

### Amyotrophic lateral sclerosis

Amyotrophic lateral sclerosis (ALS) is one of the most common forms of motor neuron disease (MND) in adults. ALS is a fatal illness that causes selective and progressive degeneration of motor neurons in the brain, brainstem, and spinal cord. Motor neuron degeneration causes muscular weakness and, in most cases, death due to respiratory failure (91). Approximately 90% of ALS cases are spontaneous, with 5-10% caused by genetic abnormalities (familial) (92).

In a recent study, the researchers discovered a variety of fungi in ALS patients’ cerebrospinal fluid and brain tissue. A number of fungus species were detected using PCR analysis and next-generation sequencing of DNA isolated from frozen brain tissue, including *Candida*, *Malassezia*, *Fusarium*, *Botrytis*, *Trichoderma*, and Cryptococcus (93). In eleven individuals with ALS, immunohistochemistry utilizing a battery of antifungal antibodies identified fungal formations such as yeast and hyphae in the motor cortex, medulla, and spinal cord (94). Recently, it has been hypothesized that the pathogenesis of ALS may be related to neurotoxins produced by the fungus (95). In response to some fungal neurotoxins, neurons increase glutamate production *in vivo*. Excessive glutamate release from neurons is one of the hallmarks of ALS, and it has been related to the death of motor neurons as a result of glutamate receptor activation (95–97). Researchers collected data from 923 U.S. counties and found a significant correlation between MND mortality and well water prevalence in those counties (98). Therefore, a hypothesis is proposed that the pathogenesis of ALS may be an opportunistic fungal infection caused by a fungus in the well water (97).

### Parkinson’s disease

Parkinson’s Disease (PD) is a progressive and debilitating disease marked by motor dysfunction and the loss of dopaminergic neurons in the substantia nigra pars compacta. This progressive loss of neurons is also associated with the release of the misfolded and aggregated protein α-synuclein (αSyn), which is expressed primarily in neurons throughout the brain (99). It has been shown that gut microbes promote the release of αSyn in mouse models and that gut microbes from PD patients impair motor function significantly more than the microbiota of healthy controls (100). One of the most noticeable aspects of PD is neuroinflammation. However, the cause is yet unknown. Neuroinflammation is mainly characterized by persistent and excessive microgliosis and astrogliosis in the substantia nigra (101). αSyn inhibits microglia autophagy extracellularly and promotes neurodegeneration in a PD mouse model (102). Knockdown of autophagy-related gene 5 (Atg5) in microglia promotes neuroinflammation and dopaminergic neurodegeneration, which is associated with the regulation of NLRP3 inflammasome activation (102, 103).

By using nested PCR analysis and next-generation sequencing to identify specific fungal species in various CNS regions from people with PD, strong evidence for polymicrobial infections in the central nervous system of PD patients is presented. Most of the identified fungal species belonged to the genera *Candida*, *Fusarium*, *Botrytis*, and *Malassezia (* 104). Recently an association between *Malassezia* and PD has been discovered (105). By searching the published literature in PubMed and EMBASE databases and analyzing the sources of heterogeneity using sensitivity analysis and meta-regression, *Malassezia* was found to be positively associated with PD risk with a significant P<0.01 (106). While *Malassezia* is the most common fungal genus in the human microbiome, over proliferation of *Malassezia* causes seborrheic dermatitis (SD), a common benign inflammatory disease of the skin. SD is prevalent in 50% of Parkinson’s disease patients but just 3% of controls (107, 108). The local skin of PD patients is characterized by an increased ratio of sebum excretion, which plays a role in SD by stimulating yeast reproduction and enzyme synthesis (108). PD risk-associated genes frequently affect lipid metabolism. Polymorphisms in GBA, PINK1, and LRRK2 (109) are three important genetic risk factors for PD, in the meanwhile increasing the concentration of intracellular lipids. *Malassezia* is a lipid-dependent fungus (110), and if *Malassezia* reaches the CNS, adequate access to lipids may allow them to colonize or over-proliferate.

Neuromelanin is present in large quantities in neurons of the substantia nigra (111). Although the exact role of neuromelanin in PD pathogenicity is unknown, neurons containing this pigment appear to die as the disease progresses (112). Unlike peripheral melanin, neuromelanin is frequently associated with lipids (113). *In vitro*, *Malassezia* was found to produce melanin from exogenous L-DOPA (the precursor of dopamine) (114), and in addition, L-DOPA triggered mycelial growth, allowing it to penetrate host cells and tissues. It is possible that *Malassezia* consumes intracellular lipid droplets from these neurons in naturally L-DOPA-rich substantia nigra to produce neuromelanin leading to exacerbation of the disease (105) (**Figure 3**). All of the above results suggested fungi may be a critical factor in neuron death and PD development. However, how to target fungi-mediated effects in PD is still to be explored.

## Conclusions

While fungi infection has been studied for a long time, the mechanism and signaling regarding the anti-fungi are still partially understood. In addition, the previous research on fungi infection mainly focuses on infectious diseases, with very limited studies on other brain disorders. In the current review, we summarized the advanced results and supported a critical role of fungi roles in CNS autoimmune and neurodegeneration disorders, such as Multiple sclerosis, Neuromyelitis optica spectrum disorder, Alzheimer’s disease, Parkinson’s disease, and Amyotrophic lateral sclerosis et al. Of note, the gut microbiota has been suggested with above CNS disorders. However, fungi have received much less attention than bacteria since they make up a smaller fraction of the human microbiome. Despite the fact that the significance of fungal infections has been highlighted in recent years, the detailed mechanism and target cells still need further exploration. In addition, the over-accumulation of fungi is also associated with specific gene mutations, such as TAGAP. The isolation and identification of fungi in the brain have revealed fresh information about the involvement of fungi in neurological illnesses. With these findings, antifungal therapy may become a potential treatment for MS and other neurological disorders.

Currently, our knowledge about the effect of fungi on CNS autoimmune and neurodegeneration-associated brain disorders is still largely unclear. Further demonstrating the role of fungi in these diseases requires in-depth studies, such as antifungal treatment of patients with neurological diseases et al.

## Author contributions

C-JZ, TP and M-LJ conceived the study. C-JZ and CW wrote the manuscript. RJ provides important suggestions for the manuscript. All authors contributed to the article and approved the submitted version.
